# Computer-assisted orbital and midfacial reconstruction

**DOI:** 10.1515/iss-2021-0035

**Published:** 2022-10-11

**Authors:** Nils-Claudius Gellrich, Fabian M. Eckstein, Björn Rahlf, Fritjof Lentge, Simon Spalthoff, Philipp Jehn, Philippe Korn

**Affiliations:** Department of Oral and Maxillofacial Surgery, Hannover Medical School, Hannover, Germany

**Keywords:** CAD/CAM, computer assisted surgery, digital planning, patient-specific implants

## Abstract

**Objective:**

Computer assistance has become indispensable in the reconstruction of the orbit and midface. Although these are key areas of an individual’s esthetic appearance, defects or deformities of the midface, especially those of the orbit, are treated diversely.

**Methods:**

The aim of this article is to present the wide utility of computer-assistance in modern craniomaxillofacial surgery, including virtual planning, computer-aided design, guided surgery, navigational control, patient-specific implants, and quality control via image fusion.

**Results:**

There have been rapid advances in both digital planning and manufacturing processes, with continual improvements.

**Conclusions:**

Patient-specific implants have pushed the boundaries of reconstructive surgery in all surgical specialties.

## Introduction

Computer assistance has a growing role in orbital and midfacial reconstruction for bony recontouring [1], [2], [3]. The orbit and midface symbolize key areas for the patients’ identity and esthetic appearance. However, there are hardly any anatomical units that are approached and treated so diversely [4]. It is globally accepted that primary and secondary, as well as congenital and acquired, deformities of the orbit and the midface are treated based on 3D imaging. Therefore, the decision to use computer assistance and implement biomedical technologies has a relatively low threshold [5]. For the last 20 years, the intensive use of robust DICOM viewers has allowed precise quantification of deformities, development of virtual blueprints for the surgical procedure, quality control process via image fusion of preoperative, intended (virtual), and postop datasets, manufacturing of patient-specific biomodels (non-corrected or corrected), and evolution of the biomaterials industry with the development of 3D printed implants with additional functionalization and preventive design elements. It has helped develop completely new protocols and approaches to correct orbital and midfacial deformities [2, 5], [6], [7], [8], [9], [10], [11], [12], [13], [14], [15], [16]. Here, we review the extensive applications of computer-assistance for enhanced surgical treatment of orbital and midfacial reconstruction.

**Figure 1:**
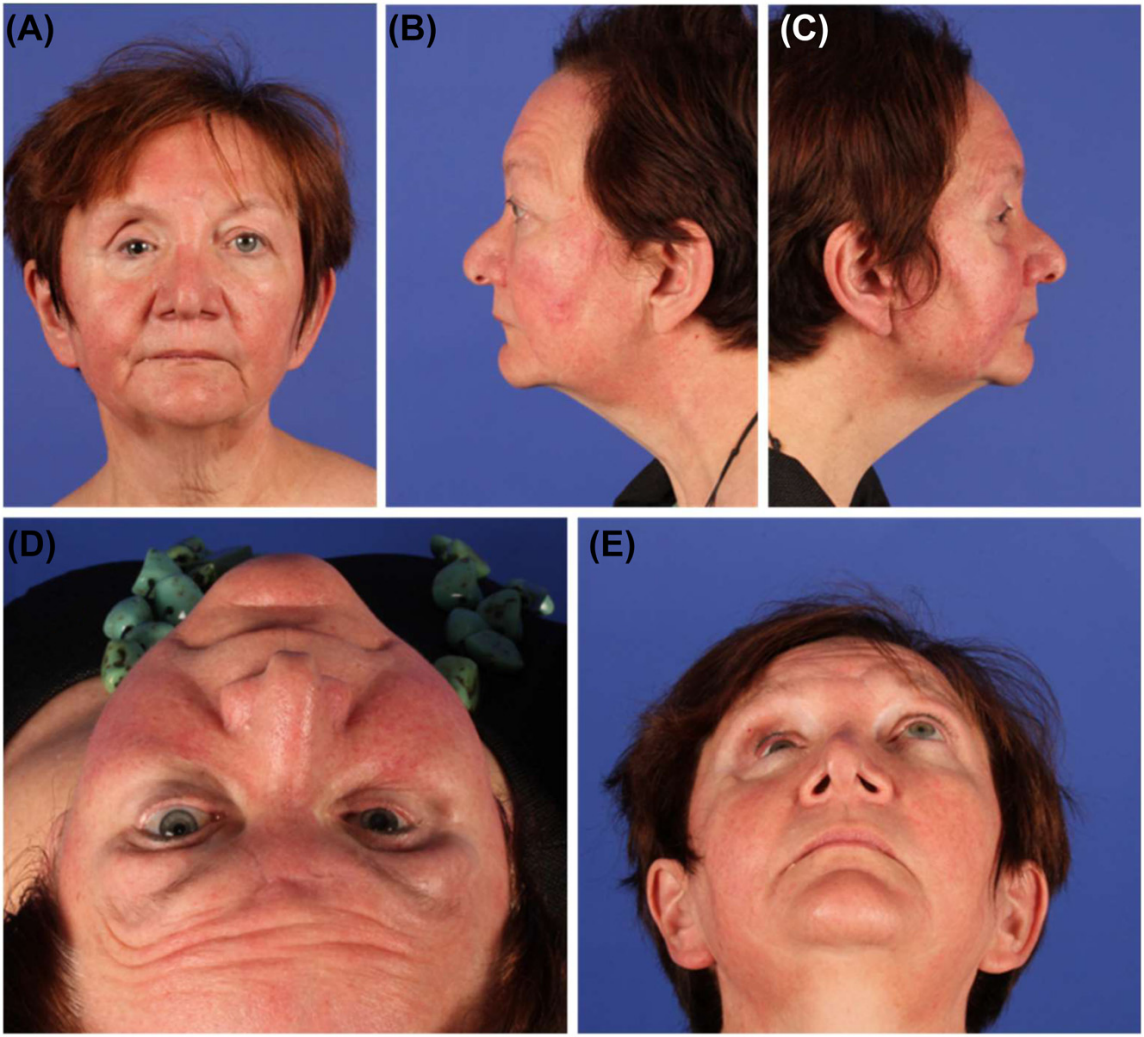
Preoperative clinical assessment of deformities: Lack of malar prominence, soft tissue sagging (A–C) with hypoglobus (A) and enophthalmos (D, E).

In cases of orbital and midfacial deformities, the first step is to evaluate the clinical features in terms of the soft tissue quality, form, and function, including examination of the mimic muscle function and thickness, elasticity, color, and texture of the soft tissues. Any deformity assessment has to be in accordance with the overall line of symmetry of the patient walking through the door. Pre-surgical assessment of symmetry must include the following clinical views: worm´s-eye, bird´s-eye, lateral, and half profile. Ideally, this view should be documented prior to any intended surgical procedure (Figure 1). For the orbits, the vertical globe position (hypo-and hyperglobus), sagittal projection (enophthalmos and exophthalmos), and eye motility should be recorded. A Naugle-exophthalmometer helps to quantify the sagittal position of the corneal projection in a side-to-side comparison, independent of the malar bone position. This clinical investigation is key because an appropriately aligned 3D dataset has to be set in comparison with the clinical situation of the individual patient. To achieve this, looking at a PACS-viewer has low to no impact on proper decision-making for orbital and midfacial reconstruction.

The following clinical indications illustrate the complexity and benefit of computer-assistance in orbital and midfacial reconstruction:

### Acquired midfacial deformity: primary orbital reconstruction

**Figure 2:**
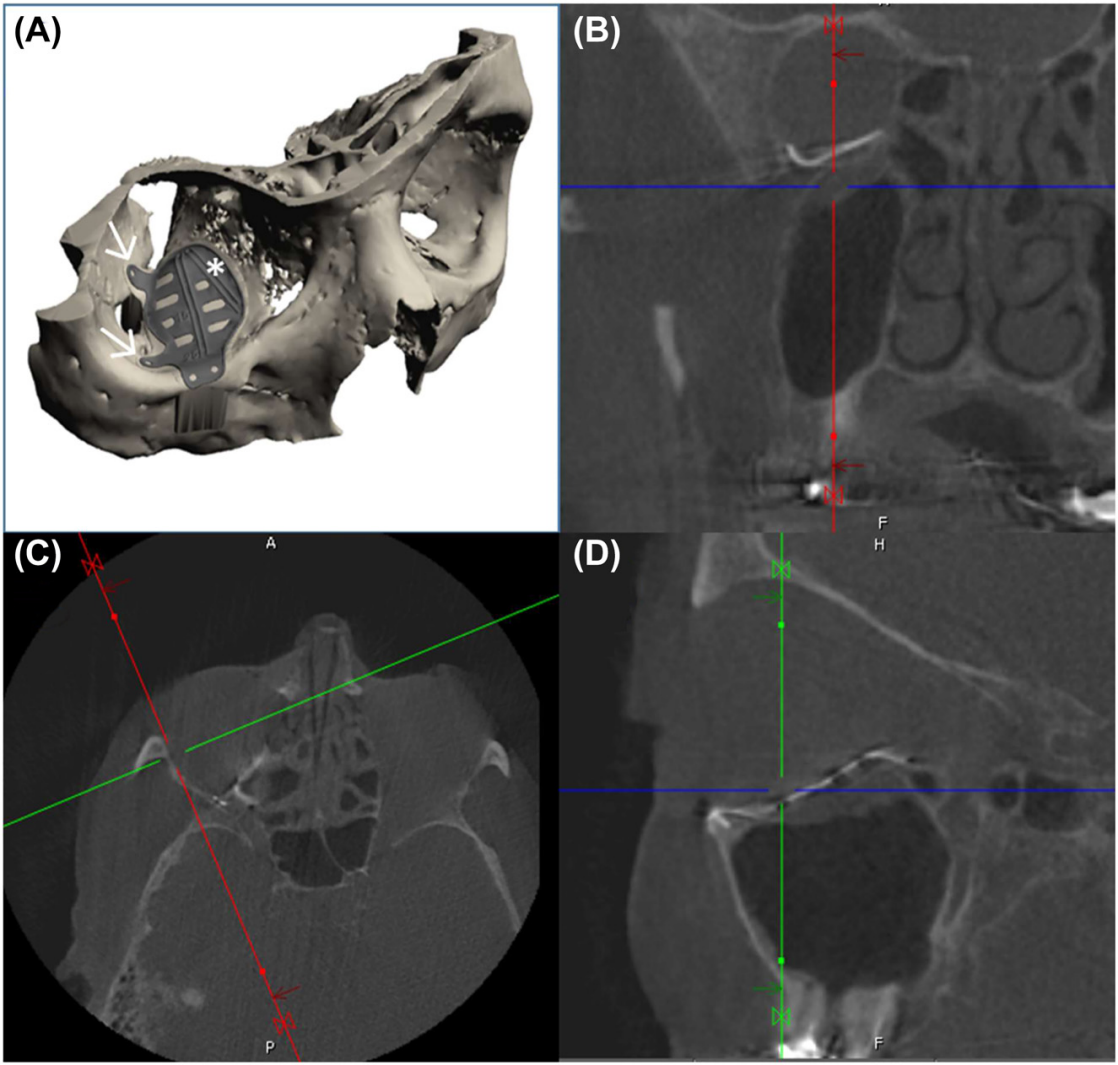
Virtual planning of orbital reconstruction (A) with a patient-specific and functionalized implant (arrows, extensions for positioning, asterisk, a trajectory) and intraoperative cone beam computed tomography after implant placement for orbital reconstruction (B–D).

After a fall accident leading to an isolated orbital floor fracture, primary treatment focused on posttraumatic deformity correction of the complete right orbital floor, starting behind the infraorbital rim up to the posterior ledge, involved prefabrication of a patient-specific implant as a one-wall orbital implant, including the transition zone, that is, the buttress between the medial orbital wall and the orbital floor. By overlapping the orbital floor defect onto the medial orbital wall, the implant can be safely placed and controlled, as shown in Figure 2. Additional design features included two extensions towards the lateral orbital wall, that is, one anterior and one in the middle, both having a center hole to allow pointer-based intraoperative navigational control plus anchorage for a 1.2 mm microscrew. The approach was fully transconjunctival and retroseptal. The patient-specific implant (IPS Implants^®^, KLS Martin, Tuttlingen, Germany) was designed and manufactured using selective laser melting technology. Further design features included two implemented vectors, which are V-shaped in a defined angle (for later trajectory based intraoperative navigation), a circular outer rounded lining around the complete implant, holds for 1.2 mm screw fixation in the infraorbital rim area, multiple openings allowing drainage into the adjacent sinuses, an inverted snow shovel design towards the posterior ledge to prevent any mechanical interference with the soft tissues close to the posterior third of the orbital cavity and the optic nerve canal entrance. Owing to the non-deformable design of the implant and the fully smooth outer lining, the implant-to-soft-tissue-interaction is less harmful than when using the available conventional orbital implants. The digital blueprint of the implant serves intraoperative quality control and can be automatically uploaded to the XYZ coordinate system of the 3D dataset of the individual patient. Pointer-based navigation can be used to ensure proper positioning of the real implant intraoperatively compared with the virtual blueprint. In addition, trajectory-based navigation allows verification of the correct vector position of the above two mentioned vectors, which allowed correct 3D object positioning of the implant in the real patient. These quality control steps ensure correct positioning of the patient-specific implant. Typically, a 1.2 mm screw is sufficient to secure the implant position. Additional landmarks to the lateral orbital wall help to self-center the implant and thus guarantee correct implant positioning. However, this is the case only if an appropriate cavity has been dissected prior to implant insertion. The postoperative 3D-data set was already acquired at the end of surgery and showed the correct and flush sitting implant position.

### Acquired midfacial deformity: secondary midfacial and orbital reconstruction

**Figure 3:**
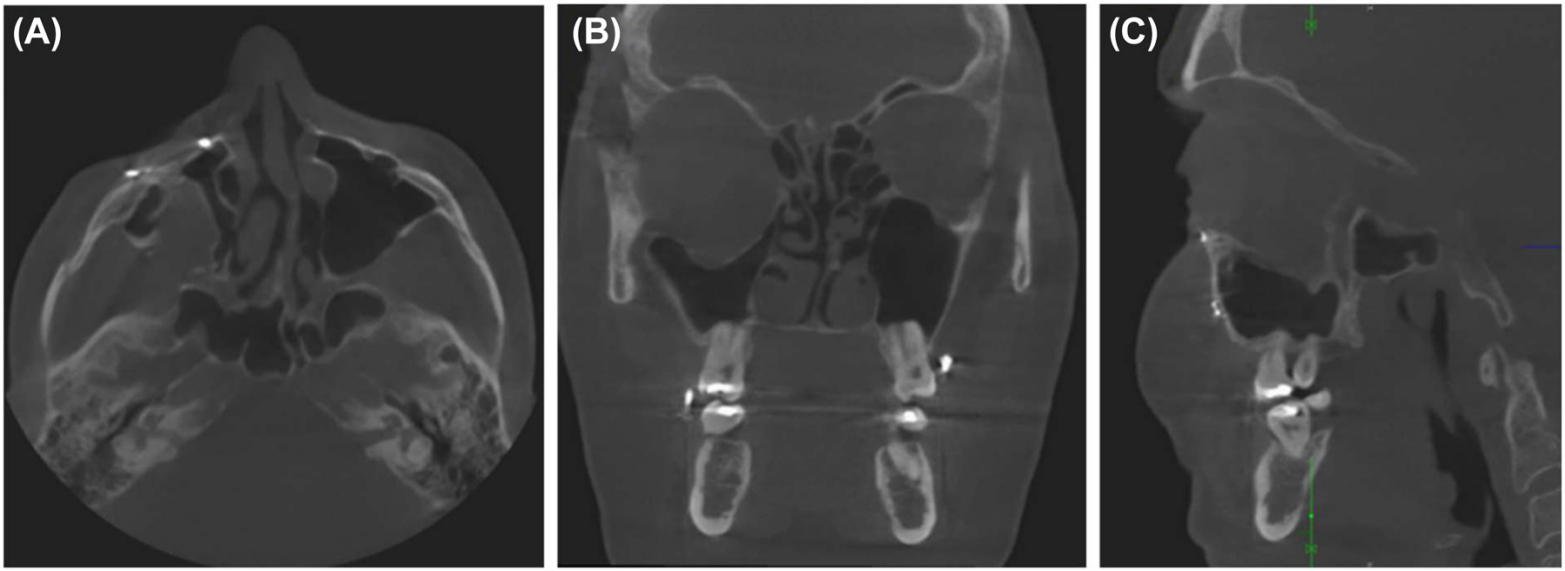
Cone beam computed tomography prior to secondary reconstruction of the patient in Figure 1. The deformity is apparent along all axes: The axial view (A) shows dislocation of the zygoma. The coronal (B) and median-sagittal (C) views show an increase in the orbital volume due to dislocation of the orbital floor.

**Figure 4:**
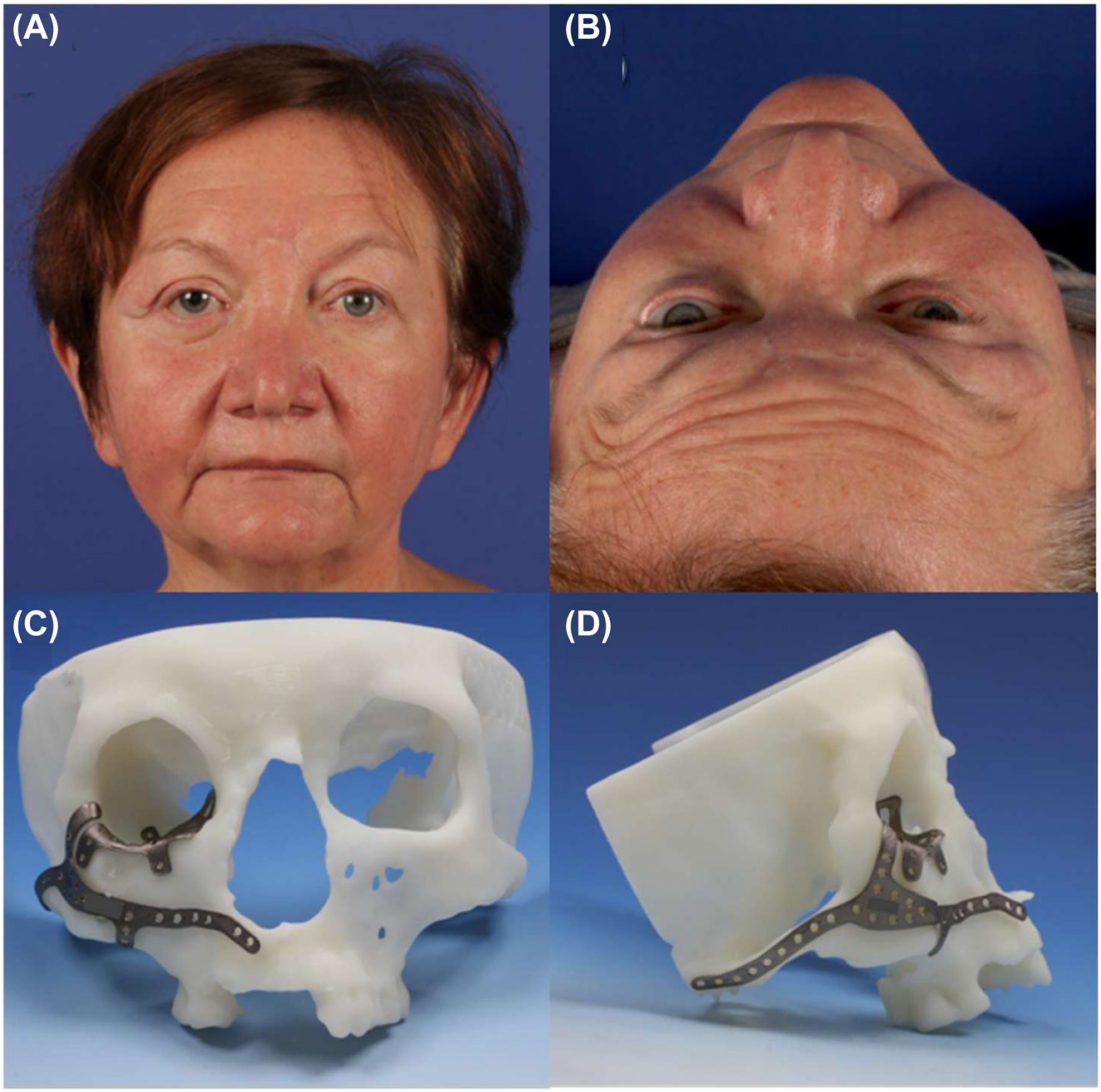
Postoperative Results following secondary reconstruction (A, B) of the patient in Figure 1 with two patient specific implants one for the outer frame and one for the orbit (C, D).

Typically, after severe car accidents, panfacial fractures occur together with closed head trauma and further extremity fractures. The following example shows the sequelae of a primary craniomaxillofacial treatment performed *alio loco*, which included repositioning of the dislocated malar bone and midface and intraorbital repair using bioresorbable implants. One year after primary treatment, the patient showed severe orbital and midface deformity, including an reduced malar prominence, midfacial soft-tissue sagging, and a severely enlarged, three-wall, right orbital volume due to projection loss of the complete orbital floor, significant hypoglobus, and enophthalmos of the right orbit (Figures 1, 3 and 4).

Computer-assisted planning involved a dual approach: one implant for the right outer frame and recontouring and the other one for the orbit (Figure 4). Both implants were designed to define the new position of the malar bone, which had to be reosteotomized and repositioned out- and forward, causing additional enlargement of the already enlarged orbital volume by changing the inner projection of the lateral orbital wall. Prior to secondary orbital and midfacial correction, significant double vision was present in all gazes; post-secondary orbital reconstruction, the patient was not compromised by double vision within the normal field of view. Intraoperative navigation was performed to ensure proper bone and implant positions. The digital design of the inner and outer implants was uploaded onto the navigation system (Brainlab^®^, München, Germany) and thus the planned implant position could be checked via navigational control within the same coordinate system of the real patient on the operating table. In addition to the three-wall patient-specific orbital implant (IPS Implant^®^ KLS Martin, Tuttlingen, Germany) titanium spacers were added to decrease the inner orbital volume, especially in the right lateral orbit. At the end of the surgery, intraoperative cone-beam CT was performed to identify and – if needed – perform any remaining corrections. All three factors together, that is, patient-specific implants, navigation and intraoperative 3D-dataset, contribute to the highest level of quality control in the field of orbital and midfacial reconstruction. Typical mistakes that might occur with these surgical corrective procedures can never be fully eliminated; however, they can be decreased to minimum.

### Acquired midfacial deformity: facial asymmetry due to posttreatment growth disturbance

**Figure 5:**
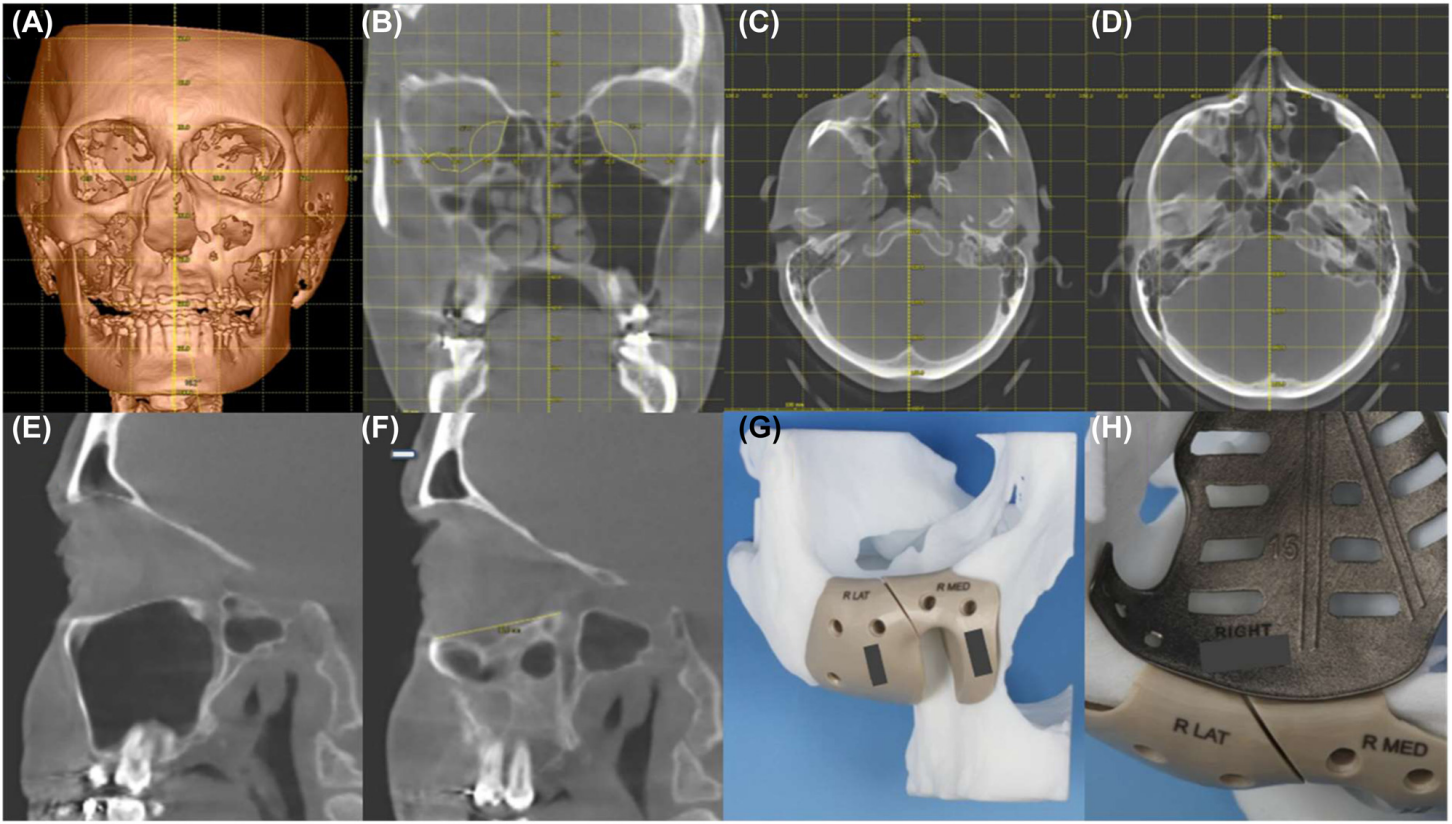
Assessment of the facial asymmetry with malar hypoplasia (A, C, and D). Growth disturbance of the right midface involves infra-positioning and thickening of the orbital floor as well as infra-positioning of the transition zone between the medial wall and orbital floor leading to an enlarged orbital volume (B). Simple application of angles in the right orbit shows the rough design of the requisite patient specific orbital implant for orbital deformity correction. The unaffected left orbit (E) is seen in the paramedian, parasagittal plane (defined by the midline of the infraorbital rim and the center of the bony optic nerve canal entrance). The corresponding sagittal view of the affected right side (F) shows an orbital deformity of over 36.3 mm, starting from the right infraorbital rim to the posterior ledge. Patient specific PEEK implants were used for malar recontouring (G) and patient specific selective laser melted titanium implant (H) for orbital reconstruction (both KLS Martin, Tuttlingen, Germany).

Trauma, infection, and radiation may cause growth disturbances. Here, we have described a case of right maxillary sinus infection and surgical treatment in early childhood. The following sequelae were clinically obvious: retruded right malar bone with less projection on the lateral midface, abnormal maxillary sinus with bony thickening of the sinus walls, orbital asymmetry with a hypoglobus and enophthalmos, and an occlusal cant. Figure 5 displays the facial deformity on different views with a superimposed reference grid for comparison with the unaffected side.

A full work-up of the facial asymmetry led to a two-step correction plan: first, correction of the projection of the right lateral and central midface as well as correction of the globe position and orbital volume. As second surgical step, bimaxillary surgery was recommended, however, the patient later refused to undergo correction of the bimaxillary complex. Nevertheless, the surgical strategy of correction, implant design, and placement described in step one include the possibility of a future Le Fort I osteotomy with a bilateral sagittal split osteotomy leading to bimaxillary correction of the facial midline.

The above-mentioned complex treatment plan led to a protocol that required two different areas of augmentation within step one: anterior maxillary sinus wall, infraorbital rim, and malar bone augmentation to provide more protection as well as orbital reconstruction to independently re-contour the aforementioned right medial wall and orbital floor defects. The inner orbital re-contouring should be handled independently of the augmentation, which is why the anchorage of the patient-specific-two-wall orbital implant was designed to be placed inside the orbit. Figure 5H displays the view of the right orbit with the patient-specific implant on a stereolithographic model, showing the two implemented vectors for trajectory-based navigation. The implant also shows the aforementioned functionalized and preventive design features, including a completely rounded outer implant margin, openings to allow for possible drainage, anatomic extensions to the lateral orbital wall and to the inside of the anterior lateral orbital rim to allow a “one-fit-only position.” To the outside, including augmentation of the infraorbital rim is a bi-parted PEEK-implant. This implant is divided into two pieces to allow safe positioning around the infraorbital nerve. Special care was taken to design the anterior midfacial implants so that Le Fort I osteotomy, including possible plate fixation, would still be feasible. The type of biomaterials for patient-specific implants and their vicinity needs special mention: inside the orbit, a metallic implant is considered excellent because of its thin build and radio-opacity for radiological control; the PEEK-implants are less thermally conductive. Fixation and position of the PEEK-implants are independent of the intra-orbital metallic implant. However, they meet each other in the area of the infraorbital rim and allow for adequate fit-control.

### Acquired midfacial deformity: postablative patient-specific orbital reconstruction and patient-specific implant-borne dental rehabilitation

**Figure 6:**
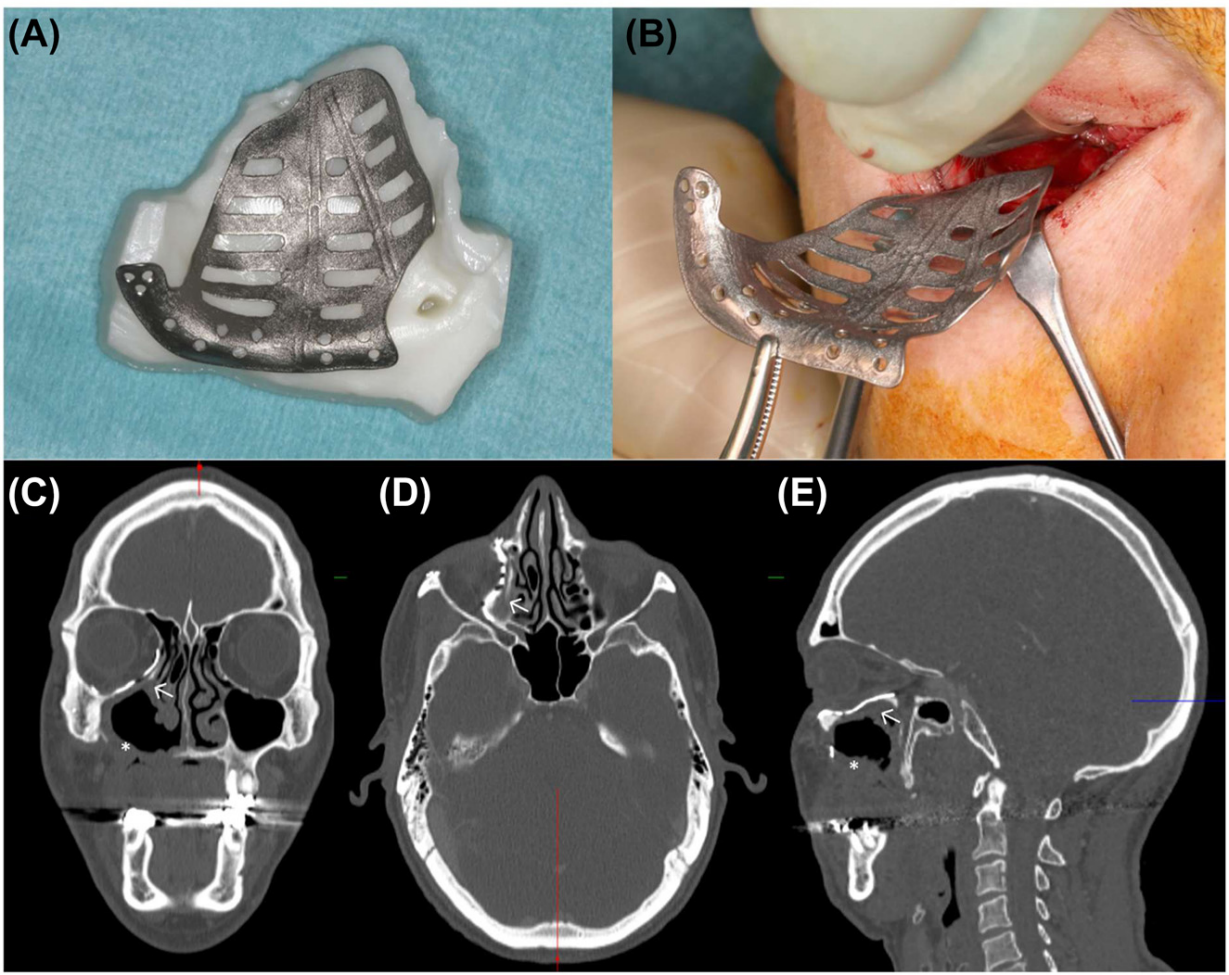
Patient-specific implant (IPS^®^, KLS Martin, Tuttlingen/Germany) on a stereolithography model (A). Implant insertion via an inferior transconjunctival approach (B). CT scan (C–E) before midface reconstruction showing orbital reconstruction with a patient-specific implant (arrows) and midface defect after hemimaxillectomy (asterisk). The complexity of the orbital reconstruction becomes apparent in the various layers: coronal (A), reconstruction of the transition zone, axial (B); reconstruction of the posterior medial bulge and median sagittal (C), reconstruction of the so-called “lazy-s” shape of the orbital floor.

**Figure 7:**
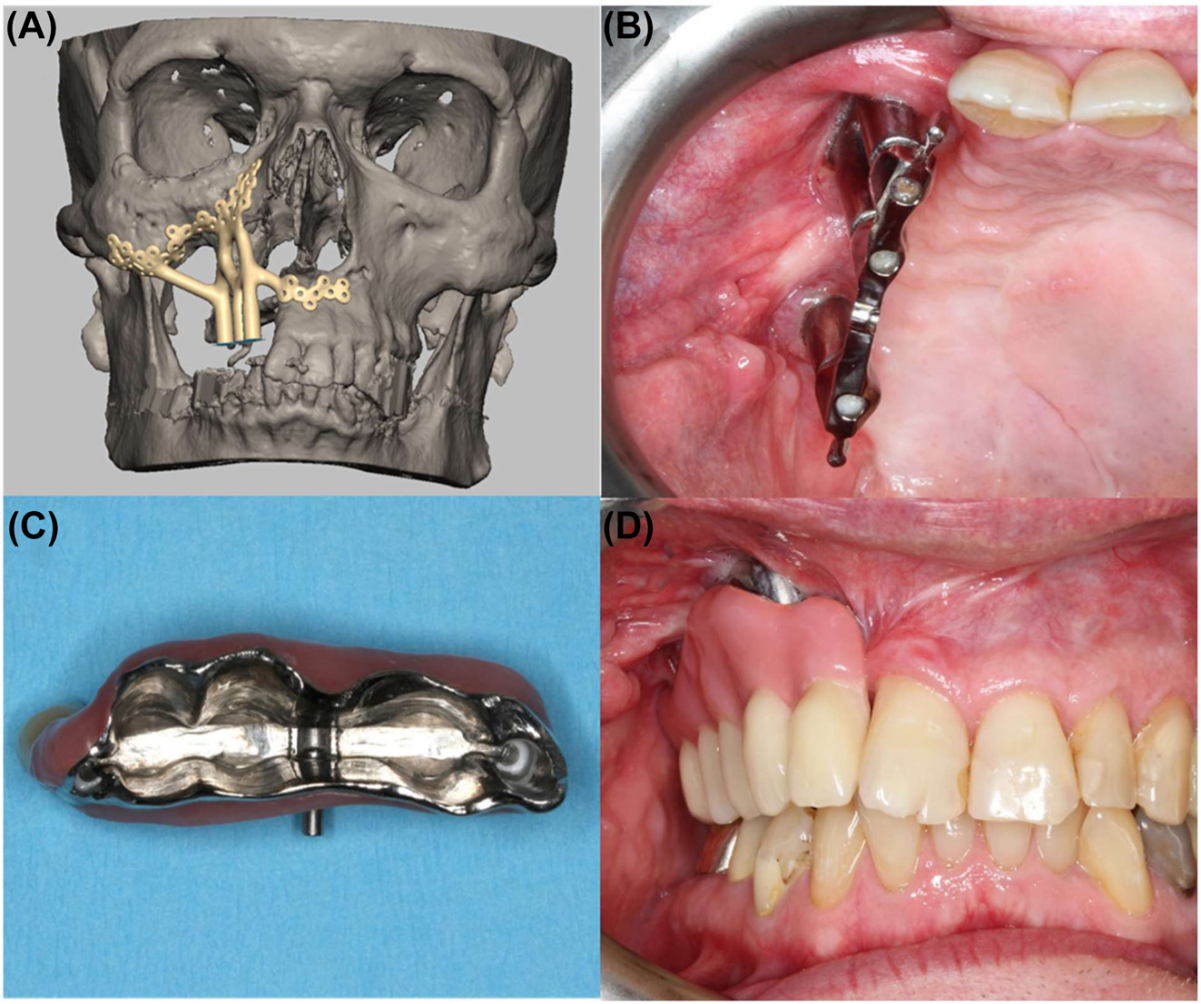
Digital planning (A) and postoperative result two years after insertion of the patient-specific implant with bar superstructure (B) and final prosthesis (C) as well as integrated dentures (D).

Ablation is one of the leading causes of acquired midfacial deformities. Here, a case of postablative extended hemimaxillary resection, including orbital floor resection, due to a benign neoplasm is discussed to demonstrate the advantage of modern computer-assisted treatment protocols during different phases. First, primary orbital reconstruction was performed using a patient-specific orbital implant (Figure 6A and B). Midfacial reconstruction with a microvascular fibular flap failed, and therefore, the maxillary defect (Brown class IIIb, Figure 6C–E) was only reconstructed by soft tissue using a microvascular lateral upper arm free flap. The second step of the original treatment protocol, that is conventional dental implant insertion, was not applicable due to bone loss. The patient was temporarily provided with a cast partial denture with clasps. During follow-up without recurrence, the patient wished to undergo functionally stable dental rehabilitation. Based on a backwards planning protocol, a patient-specific one-piece implant based on a metallic framework mounted with three posts was digitally designed and manufactured using selective laser melting technology (IPS Implants^®^ Preprosthetic, KLS Martin, Tuttlingen, Germany, Figure 7A). The implant was fully loadable directly following insertion in an outpatient setting. The patient was therefore first provided with a provisional prosthesis and then the final prosthesis after 3–6 months. Multivector-anchorage with multiple 1.5 to 2.0 miniscrews ranging from to 5–13 mm in length allows for rigid fixation away from the transition zone of the posts with soft-tissue coverage, which is significantly different from conventional dental implants. The latter relies on bone for anchorage with a minimum of 1.5 mm of vital bone around the implant geometry; the IPS preprosthetic, due to its distant anchoring principle, is independent of the bone close to the aforementioned soft tissue to the implant shoulder interface. Even very distant anchorage onto the pterygoid process or into the lateral skull base is feasible, provided there is a lack in the midfacial region.

This innovative approach using computer-assisted planning and manufacturing has made it rapid dental rehabilitation possible without requiring time-consuming hard and soft tissue transfers that add to the stress of the patient. Figure 7B–D shows the implant *in situ* with the bar-supported prostheses and final postoperative results.

### Acquired midfacial deformity: postablative reconstruction with guided surgery for implant-borne epithesis

**Figure 8:**
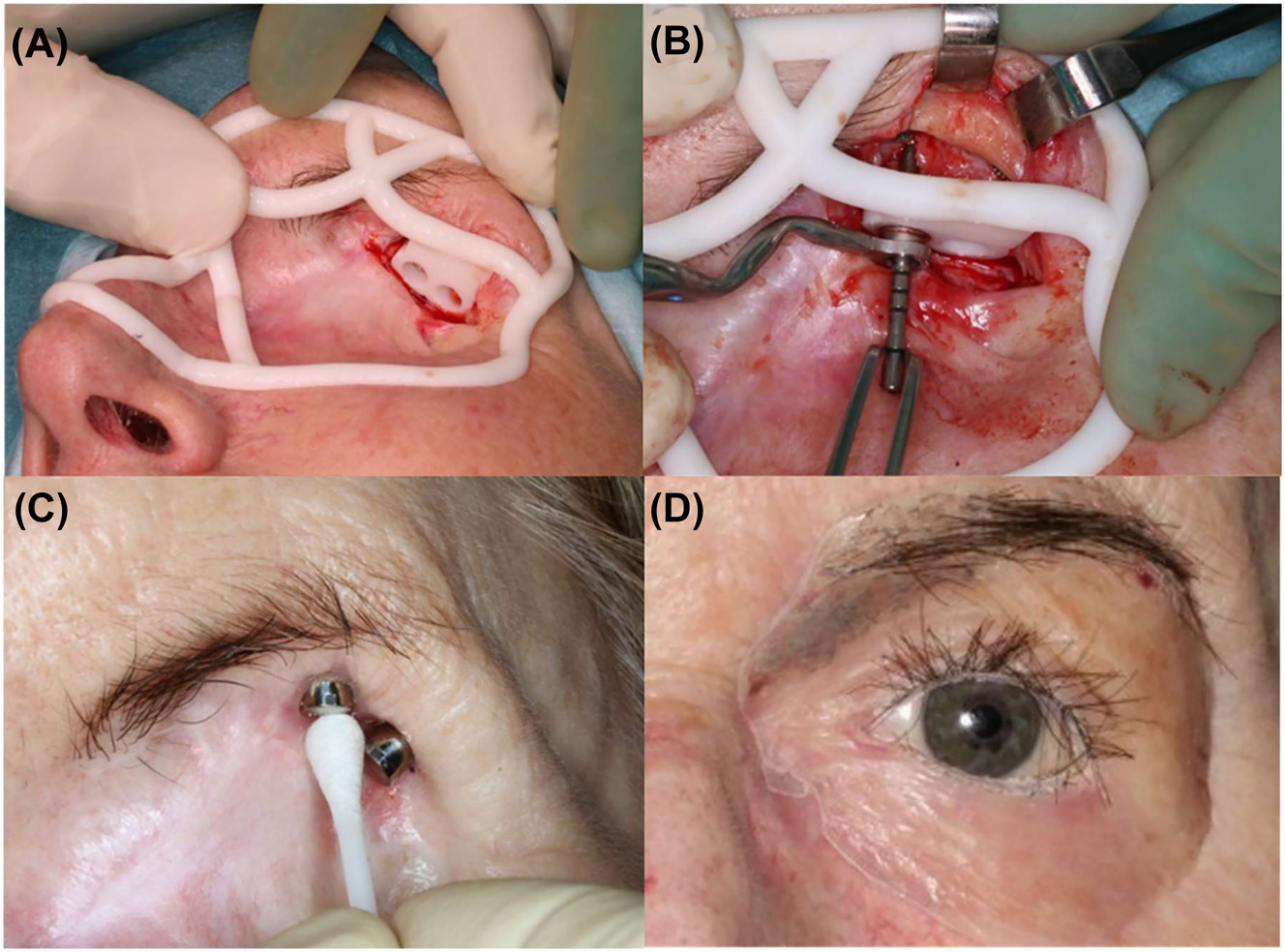
CAD/CAM drill guide (A) for implant insertion (B) and postoperative results (C) as well as prosthesis (D).

Computer-assistance is not only versatile in the detection and resection of malignant tumors but may contribute significantly to the quality of life after tumor treatment. This is demonstrated in a case of conjunctival carcinoma, in which multiple R1 resections had been performed at a different hospital. The oncological therapeutic concept had to be extended towards orbital exenteration of the left orbit, including ablation of the upper and lower eyelids. Primary defect reconstruction included a pedicled temporalis flap for the orbit; to camouflage the left temporal region as the donor site, a 3D-titanium mesh was contoured and retained with screws to prevent temporal hollowing. Eighteen months after ablation and reconstruction of the left orbit, two periorbital implants (Straumann, Basel, Switzerland) were inserted using a CAD/CAM drill guide according to the guided surgery protocol, which is, using the drill handles with cylinders inserted into the generic sleeve design of the soft tissue-borne drill guide. The drill guide was digitally designed and manufactured via 3-D printing technology using an autoclavable resin. The complex and extended 3D-design of the latter allowed a perfect soft-tissue surface of the drill-guide interface and appropriate placement without requiring pin fixation. Due to the limited amount of bone for anchorage, only two short extraoral implants could be placed; further limitations in the design of the epithesis were related to the patient´s desire to retain her left eyebrow. Figure 8 demonstrates the precise fit of the implant shoulder position following backwards planning and quality control by the combined use of a patient-specific drill guide with the measuring gauge and the final implant-borne prosthesis.

## Discussion

Computer assistance in craniomaxillofacial surgery, especially in orbital and midfacial reconstruction, has a special impact on pre-, intra-, and postoperative quality control, especially in complex procedures [2, 6, 11]. In fact, it is the anatomical regions for which the clinical applications of computer-assistance became of special interest [17]. A 3 D volume dataset can be used for interactive analysis, virtual modeling (i.e., mirroring, segmentation, image fusion, etc.), communication within the specialty or out of the specialty, with the patient, for medicolegal documentation, and for under- or post-graduate training [18, 19]. A higher level of transparency is guaranteed and allows an evidence-based approach for each individual case by quantifying deformities and foreseeing, controlling, and validating surgical steps, especially for hard tissue-based corrections. To achieve this, the quality of a diagnostically indicated spiral CT or cone beam CT scan has to meet the basic criteria, such as a minimum slice thickness of 0.6–1 mm, and DICOM data have to be exported [20]. In routine clinical practice, poor quality of diagnostic datasets coming with referred patients for consultation is the main reason for extra scanning needs for 3D-preoperative datasets.

The first clinically convincing protocol to use 3D-datasets was developed for craniofacial defects and placement of CAD CAM implants in the area of the cranial vault. Here, the scientific group around H. Eufinger and E. Machtens has to be given credit for their huge achievements in defining the digital workflow to digitally design and manufacture patient-specific implants [21], [22], [23]. In those days, CNC milling was the main way to produce these implants; however, this implies certain restrictions for the design. Today, it is mainly based on selective laser melting technology and additive manufacturing, which allows any type of design for manufacturing the inner and outer structures of an implant [16]. In the case of the cranial vault, accuracy is less important for recontouring; however, it is of upmost importance if it comes to reconstruction in and around the orbit and midface. Out of the challenging orbital region, evolutionary redesigning ideas were started for patient-specific implants. This is due to the fact that not only the orbital contents is vulnerable and the shape of the orbit is demanding for recontouring, but if it comes to function, there is hardly a more complex region in the human body than the orbit itself, where six out of 12 cranial nerves in humans deal with this volumetric-wise small area of the human body. During dissection, exploration, implant insertion and repositioning of soft tissues several pitfalls might occur leading to major functional problems like double or impaired vision up to blindness, not to mention disfigurement. This is why computer assistance could help to close the gap between the surgeon’s plan and demands and pre- and postoperative 3D-scanning of the patient. The aim was to raise biomedical implants above the level of recontouring of what has been there: functionalization of patient-specific orbital implants in a preventive design started and lead to new innovative implant designs [3, 15, 16]. As a result, implants became reliable elements to treat defects and deformities via smaller approaches (mainly transconjunctivally for the orbit), to less invasively insert the implant (due to trimmed design and circular rounding) that is stiff enough to not deform during insertion and has additional design features like the inverted snow-shovel design to slip onto the posterior ledge and reliably point away from the tissues close to the optic canal, to visually control the correct fit (extensions for positioning and landmarks) via navigation of the correct object position (point-based or trajectory-based), and to provide proof treatment (radiopacity) with little need for extra-fixation, which in orbital implants can mainly be achieved with one screw only.

Thus, computer assistance leads to a sound protocol for the management of orbital deformities with quality control: from virtual planning to the navigational control of preoperatively designed digital blue-prints. The combination of patient-specific implants and intraoperative imaging computer-assistance in the orbital region defines the highest level of surgical quality control at present.

Earlier, it was mainly Joseph Gruss, who advocated the importance of recontouring the outer midfacial frame [24]. The orbit defines the appearance around the globe, and the outer frame is crucial in defining skeletal facial symmetry. It is a huge advantage of computer assistance that it enables planning and treatment of severe midfacial deformities to design a flush sitting outer implant running from the lateral skull base to the subnasal area over the malar prominence to define a hardware-based contour of the lateral midface. In case of multipiece fractures or the need for re-osteotomy of malar bones healed in malposition, patient-specific implants can be inserted transorally in combination with an upper pre-auricular approach and clearly define the new position of the segments that can be screw retained from the inside to his outer-frame implant. Furthermore, additional landmarks may help in better positioning or provide further bone segment position control. Even different biomaterials (i.e., titanium and PEEK) can be combined for different implants or implants can be split to ease insertion if required.

New implants and designs have been developed thanks to computer assistance and digital planning [25]. Here, we have described the use of computer assistance to address the need for dental rehabilitation in case of major bone loss and minimally invasive and quick restoration of the chewing function [26], [27], [28]. The limitation for the new implant, IPS preprosthetic^®^, was hardly on the level of bone but on the level of soft tissues, which need adequate quantity and quality to allow separate anatomical units to function. Today, this line extension to conventional dental implants or even special implants such as zygomaticus fixtures allow for complex implant-borne prosthodontic restorations either in the maxilla or mandible. However, the maxilla is important in this regard because unfavorable biomechanical situations typically develop in the maxilla, for example, as an Angle class III relationship due to atrophy, growth disturbance, or tissue loss, where the pneumatized regions of the nose and maxillary sinuses and the highly mobile tissues around the lip and cheek define the boundaries for treatment options. Here, it was a major advantage to design a perfectly designed one-piece implant that can be inserted during one outpatient-based surgery with multivector screw retention as a primarily and functionally stable patient-specific implant with no extra need for abutment adjustment due to perfect alignment and parallelization of the implant posts manufactured on the basis framework by laser melting technology out of titanium alloy metal powder via additive manufacturing. Even the weak spot of dental implants could be overcome, that is, the transition from soft tissue to implant shoulder and bone might be the point of entry for peri-implantitis, leading to compromised bone anchorage and later implant loss. Owing to the distant anchorage of the IPS Implant^®^ Preprosthetic onto bony structures that are reliable (e.g., medial and lateral buttresses of the midface, anterior nasal spine), screw lengths of 5–13 mm can be used to rigidly fixate a maxillary implant for both sides in edentulous patients with a mean of 20 screws. Careful soft tissue management includes the shielding of the posts in the lateral maxilla via the pedicled Bichat buccal fat pad. To date, we have completed 63 complex IPS Implant^®^ Preprosthetic implant insertions, in which the power of this new technology became obvious, especially in desperate cases of failure of previously applied conventional treatment protocols. Not limited to cases of IPS Implants^®^, pre-prosthetic 3D-printed guides might be helpful in pre contouring the recipient site and harvesting of bone grafts, to guide osteotomies or to position- and vector control endosseous implants, they might be of great help during surgery. Instead of bone anchoring, only more extended guide designs help to accurately position the 3D-guides manufactured out of polyamide, even in cases of atypical anatomy. Guided surgery has become quite common in advanced dental implant treatment; however, in midfacial reconstruction, their importance is increasing. The advantage is that even modern 3D-printing allows the fabrication of these guides or biomodels with autoclavable resins in-house of the medical institution.

Today, individualization in craniomaxillofacial surgery is mainly related to computer assistance and its application during surgical treatment. The big advantage is that computer assistance helped quantify surgical results pre-, intra-, and post-operatively and thus significantly contributed towards quality control in the creation of evidence on the level of the individual patient. Here, orbital and midfacial reconstructions were and are the major driving forces.

## Supplementary Material

Supplementary Material
