# Supplementary material for: Computer-assisted orbital and midfacial reconstruction
Source: Innov Surg Sci. 2022 Oct 11;8(3):185–94. doi: 10.1515/iss-2021-0035 (PMC10709693; doi:10.1515/iss-2021-0035)
Supplement: Supplementary file 1 — Supplementary Material [file j_iss-2021-0035_suppl.pdf]

Reviewer Assessment

N.-C. Gellrich et al.: Computer-assisted orbital and midfacial reconstruction

Reviewers' Comments to Original Submission

Reviewer 1: Parmar, Sat

Date received: 4-Mar-2022
Reviewer recommendation: **Accept in present form**
Reviewer overall scoring: **Excellent**

Assessment Form scores: 5 = High/Yes; 3 = Medium/Adequate; 1 = Low

Is the subject area appropriate for the journal	5				
Does the title clearly reflect the paper's content?	5				
Does the abstract clearly reflect the paper's content	5				
Do the keywords clearly reflect the paper's content?	5				
Does the introduction present the problem clearly?	5				
Are the results/ conclusions justified?		4			
How comprehensive and up-to-date is the subject matter presented?	5				
How adequate is the data presentation?		4			
Are units and terminology used correctly?					
Is the number of cases adequate?		4			
Are the experimental methods/ clinical studies adequate?		4			
Is the length appropriate in relation to the content?		4			
Does the reader get new insights from the article?		4			
Please rate the practical significance.	5				
Please rate the accuracy of methods.		4			
Please rate the statistical evaluation and quality control.			3		
Please rate the appropriateness of the figures and tables.	5				
Please rate the appropriateness of the references.	5				
Please evaluate the writing style and use of language.		4			
Please judge the overall scientific quality of the manuscript.	5				
Are the methods used worthy of reproduction in greater deal?	Yes				
Would you be willing to review a revision of this manuscript?	Yes				

Comments to author: Well written with good pictures and results.

Reviewer 2: Schramm, Alexander

Date received: 03-Mar-2022
Reviewer recommendation: **Return to author for minor modifications**
Reviewer overall scoring: **High**

Assessment Form scores: 5 = High/Yes; 3 = Medium/Adequate; 1 = Low

Is the subject area appropriate for the journal	5				
Does the title clearly reflect the paper's content?	5				
Does the abstract clearly reflect the paper's content	5				
Do the keywords clearly reflect the paper's content?	5				
Does the introduction present the problem clearly?	5				
Are the results/ conclusions justified?	5				
How comprehensive and up-to-date is the subject matter presented?		4			
How adequate is the data presentation?	5				
Are units and terminology used correctly?	5				
Is the number of cases adequate?	5				
Are the experimental methods/ clinical studies adequate?	5				
Is the length appropriate in relation to the content?	5				
Does the reader get new insights from the article?	5				
Please rate the practical significance.	5				
Please rate the accuracy of methods.	5				
Please rate the statistical evaluation and quality control.		4			
Please rate the appropriateness of the figures and tables.	5				
Please rate the appropriateness of the references.	5				
Please evaluate the writing style and use of language.		4			
Please judge the overall scientific quality of the manuscript.	5				
Are the methods used worthy of reproduction in greater deal?	Yes				
Would you be willing to review a revision of this manuscript?	Yes				

Comments to author: Congratulations to this nice case serious. It si really an excellent up to date of todays possibilities in computer assisted orbital and periorbital reconstruction. Ref. 9 and 18 are the same, please correct.

Authors' Response to Reviewer Comments

Date received: 31-May-2022

Response to reviewer 1

Comments to the Author
Congratulations to this nice case serious. It si really an excellent up to date of todays possibilities in computer assisted orbital and periorbital reconstruction. Ref. 9 and 18 are the same, please correct.

Response: As per your comment we have changed Ref. 18 to the following:

Gellrich NC, Rahlf B, Jehn P, Spalthoff S, Korn P. Outcome and Quality of Life after Individual Computer-Assisted Reconstruction of the Midface. Laryngorhinootologie. 2022 May;101(S 01):S90-S102. English, German. Risk

Response to reviewer 2

Comments to the Author
Well written with good pictures and results

Response: Thank you for your kind comment. No changes are required

Comments by the Editor-in-Chief to Revised Submission

All reviewer comments were addressed adequately and the manuscript should be published in its present stage.
